# Bilateral Carotid Artery Dissection Due to Asymmetric Styloid Processes: A Rare Case Report Highlighting the Role of Anatomical Factors Beyond Length

**DOI:** 10.7759/cureus.90185

**Published:** 2025-08-15

**Authors:** Kazushi Kitamura, Koichi Iwasaki, Kazuyuki Mikami, Hiroshi Hasegawa, Isao Sasaki

**Affiliations:** 1 Neurosurgery, Ainomiyako Neurosurgery Hospital, Osaka, JPN

**Keywords:** bilateral carotid artery dissection, carotid artery stenting (cas), eagle syndrome, stroke, stylocarotid artery syndrome, styloid ligament, styloid process, transcervical styloidectomy

## Abstract

Elongation of the styloid process (SP) is a recognized etiologic factor in vascular pathologies such as carotid artery dissection (CAD), particularly in Eagle syndrome or stylocarotid artery syndrome. However, CAD involving an SP of normal length is exceedingly rare and often overlooked in clinical evaluations. We present a unique case of bilateral CAD caused by mechanical injury from asymmetric SPs - one elongated and the other of normal length - challenging the conventional belief that only elongated SPs carry clinical importance.

A 47-year-old female patient experienced sequential episodes of bilateral CAD over a seven-month period. Radiological evaluations identified bilateral CAD likely attributed to the left SP of normal length (22 mm) and the right SP with elongation (35 mm). Both SPs were located in close proximity (<1 mm) to their respective internal carotid arteries (ICAs). Notably, despite its normal length, the left SP had a steep medial angulation of 65.1°, contributing to the ICA impingement. To restore vascular patency and prevent recurrence, the patient underwent successful bilateral carotid artery stenting followed by transcervical styloidectomies.

While elongated SPs are commonly implicated in unilateral vascular symptoms, bilateral CAD related to SP impingement is rare. This case illustrates that even an SP of normal length can cause vascular injury when it exhibits atypical angulation and lies in close proximity to the ICA. Contrary to the conventional belief that only SP elongation has clinical importance, SPs of normal length may play a pathogenic role in SP-related CAD under specific anatomical conditions. We emphasize the importance of a comprehensive radiological evaluation that focuses not solely on SP length when considering potential SP-related vascular pathology.

## Introduction

Eagle syndrome is a rare clinical disorder characterized by neuropathic and/or vascular symptoms caused by an elongated styloid process (SP) or a calcified stylohyoid ligament [1-3]. Although SP elongation is observed in approximately 4-10% of the general population, only a small proportion of these individuals develop symptoms [1-3]. Among these, vascular Eagle syndrome, a subtype presenting exclusively with vascular symptoms, accounts for about 23.5% of all Eagle syndrome cases [2, 4]. A closely related entity, stylocarotid artery syndrome (SCAS), also involves SP-related vascular compression [5, 6]. Although vascular Eagle syndrome and SCAS are thought to represent a spectrum of the same disease, they may differ in the diagnostic criteria [5, 6]. While Eagle syndrome is traditionally defined by a SP length of 30mm or greater [2, 3], SCAS does not necessarily require a specific SP length threshold for diagnosis [5 - 7]. SCAS may occur even with SPs of normal length, particularly in the presence of other anatomical variations such as steep medial angulation, aberrant spatial orientation, or close proximity of the SP to the carotid artery [7-9]. These variations can predispose patients to vascular complications, including carotid artery dissection (CAD) [7-9].

SP-related vascular symptoms are generally unilateral, and bilateral CAD attributed to SP compression is exceedingly rare [10-14]. Here, we present a unique case of bilateral CAD caused by asymmetric SPs, one elongated and the other within normal anatomical length. The patient exhibited specific anatomical variations that likely contributed to the development of CAD. This case highlights the importance of assessing not only SP length but also its spatial orientation and anatomical relationship to adjacent vessels when investigating the pathophysiology of unexplained CAD. To our knowledge, bilateral CAD involving an SP of normal length has rarely been reported.

## Case presentation

**Figure 1:**
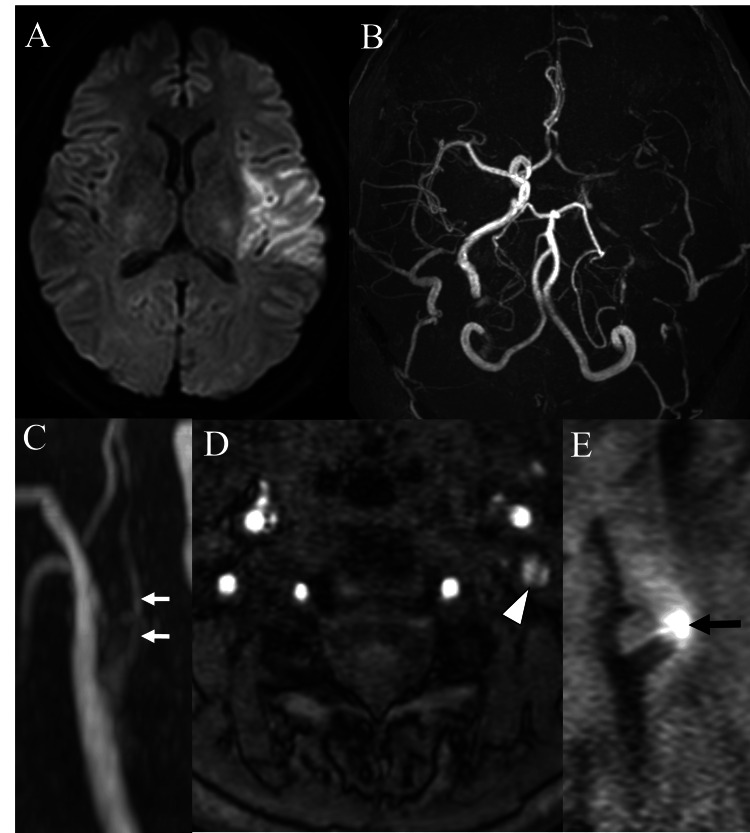
Radiological images at the initial presentation. Diffusion-weighted magnetic resonance (MR) image showing a hyperintense area in the left temporal lobe, indicative of acute infarction (A). Brain MR angiography (MRA) demonstrating reduced signal intensity in the left middle cerebral artery territory (B). Cervical MRA showing near occlusion of the left internal carotid artery (white arrows) with an associated intimal flap (white arrowhead) (C, D). T1-weighted black-blood MRI demonstrating a high signal intensity area in the left ICA, consistent with an intimal hematoma (black arrow) (E). ICA: internal carotid artery; MCA: middle cerebral artery

A 47-year-old woman experienced bilateral CAD, occurring sequentially over a seven-month period. Her medical history was unremarkable with no evidence of underlying vascular or connective tissue disorders, such as fibromuscular dysplasia or Ehlers-Danlos syndrome. She also reported no history of trauma, sudden neck movements, or vigorous physical activity. At her first presentation to the emergency department, the patient presented with sudden-onset aphasia and right hemiparesis, with a National Institutes of Health Stroke Scale (NIHSS) score of 21. Diffusion-weighted magnetic resonance (MR) imaging showed an acute infarction in the left temporal lobe (Figure 1A). Brain and cervical MR angiography (MRA) demonstrated signal reduction in the left middle cerebral artery (MCA) territory and severe stenosis of the left ICA with an associated intimal flap (Figures 1B-1D). T1-weighted black-blood MR imaging confirmed high signal intensity within the left ICA, suggesting an intimal hematoma (Figure 1E).

**Figure 2:**
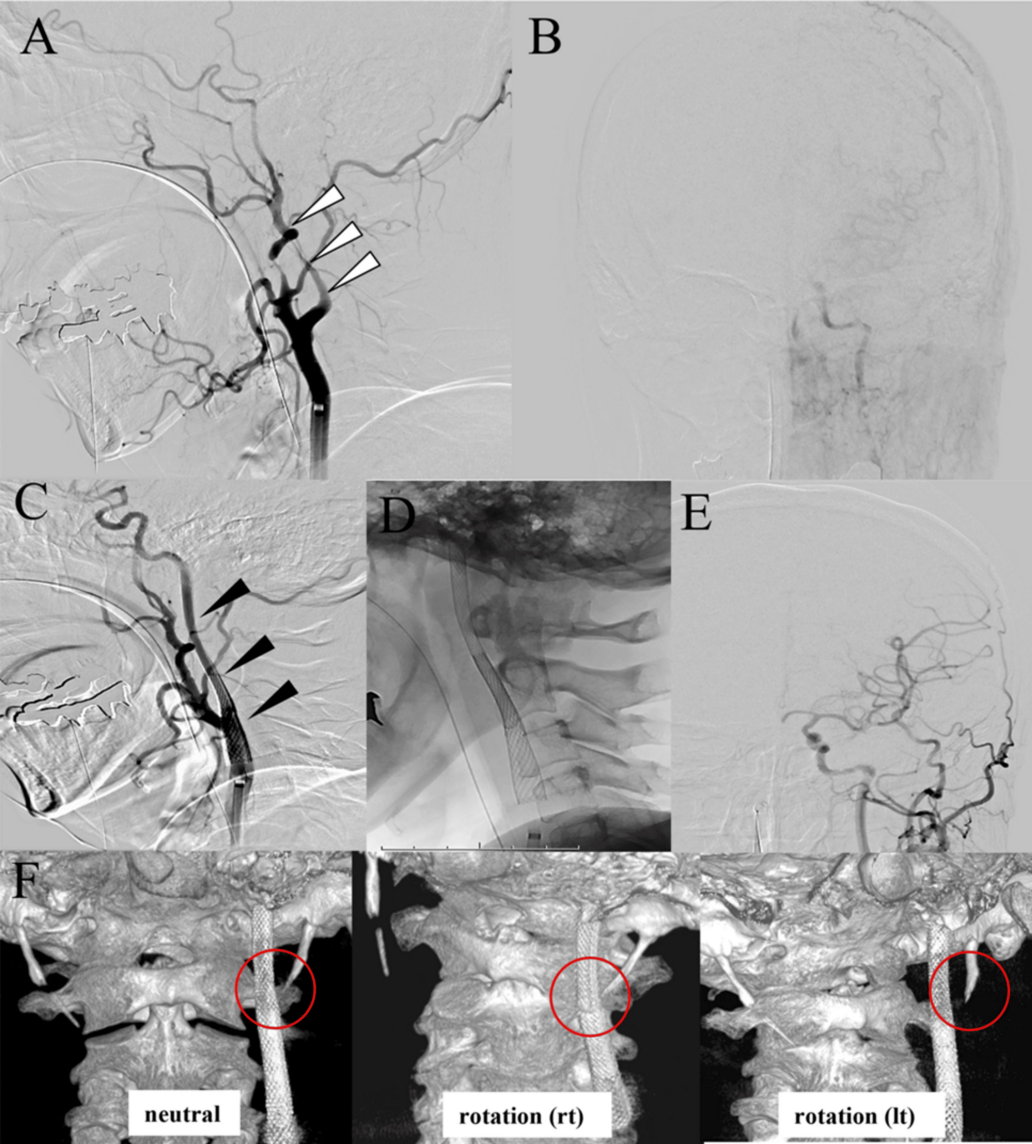
Digital subtraction angiography and cone-beam CT with dynamic head rotation during the initial intervention. Preoperative images showing near occlusion of the left ICA (A) with reduced blood flow in the left MCA territory (B). Postoperative images demonstrating successful CAS with restored patency of the left ICA (C), the stent shadow visible in the non-subtracted image (D), and improved perfusion in the left MCA territory (E). Cone-beam CT in dynamic head positions (left: neutral, center: rightward rotation, right: leftward rotation), revealing a close and variable spatial relationship between the left SP and ICA with head rotation (highlighted in red circles) (F). Note that the SP is observed to directly impinge on the ICA at rightward rotation. CAS: carotid artery stenting; ICA: internal carotid artery; MCA: middle cerebral artery; SP: styloid process

Suspecting left ICA dissection resulting in flow reduction of the left MCA, emergent carotid artery stenting (CAS) was performed. Digital subtraction angiography (DSA) during the intervention confirmed near complete occlusion of the left ICA with reduced blood flow in the left MCA territory (Figures 2A-2B). As the precise location of the dissection could not be determined, stent deployment was performed to cover the entire segment from the intracranial portion to the cervical portion of the left ICA. The CAS procedure began with the introduction of a 9Fr OPTIMO balloon guide catheter (Tokai Medical Products, Aichi, Japan). To secure access to the true lumen of the left ICA, an SL-10 microcatheter (Stryker, Fremont, CA, USA) was carefully navigated across the lesion. Subsequently, left CAS was performed via the SL-10 microcatheter to reconstruct the affected segment. A total of four stents were deployed in sequence: two Neuroform Atlas stents (4 × 40 mm, Stryker, Fremont, CA, USA), one Carotid Wallstent (8 × 29 mm, Boston Scientific, Marlborough, Delaware, USA), one Carotid Wallstent (10 × 31 mm, Boston Scientific, Marlborough, Delaware, USA). The procedure successfully restored patency of the ICA and improved perfusion in the left MCA territory (Figures 2C-2E). Dynamic imaging with head rotation using cone-beam CT was conducted during the intervention. The study revealed a close and variable spatial relationship between the left SP and ICA with head rotation, consistent with a diagnosis of left-sided SCAS (Figure 2F). The patient’s postoperative course was uneventful, with gradual resolution of her neurological deficits. She presented with right hemiparesis and aphasia at the early post-procedure period with an NIHSS score of 20. However, she was discharged home one month later with an NIHSS score of 0.

**Figure 3:**
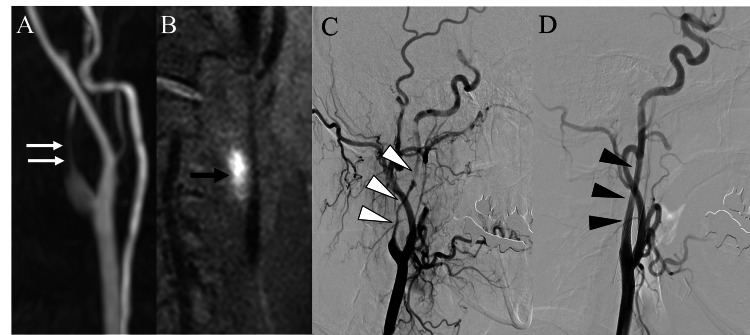
Radiological images at the second presentation. A: Cervical MRA showing near occlusion of the right ICA (white arrows). B: T1-weighted black-blood MRI demonstrating a high signal intensity area in the right ICA, consistent with an intimal hematoma (black arrow). C: Preoperative DSA showing severe stenosis of the right ICA (white arrowheads). D: Postoperative DSA demonstrating successful CAS with restored patency of the right ICA (black arrowheads). ICA: internal carotid artery

Seven months after the onset of the left CAD, the patient returned to the emergency department with a complaint of occipital headache, but no neurological deficits were observed. Although diffusion-weighted MR imaging showed no signs of cerebral infarction, MRA revealed severe stenosis of the right ICA (Figure 3A). T1-weighted black-blood MR imaging confirmed high signal intensity in the right ICA, suggesting an intimal hematoma related to the right ICA dissection (Figure 3B). Given the potential risk of cerebral infarction, emergent CAS was performed to prevent further ischemic events. DSA during the intervention confirmed severe stenosis of the right ICA (Figure 3C). Due to a suspected thrombus in the petrous portion of the right ICA, mechanical thrombectomy was initially performed. An 8Fr OPTIMO balloon guide catheter (Tokai Medical Products, Aichi, Japan), a Catalyst 6 aspiration catheter (Stryker, Fremont, CA, USA), and an SL-10 microcatheter (Stryker, Fremont, CA, USA) were used for this purpose, and a red thrombus was successfully aspirated. Subsequently, a right CAS was performed to reconstruct the affected segment of the right ICA. Three stents were deployed in sequence: one Wingspan stent (3.5 × 20 mm, Stryker, Fremont, CA, USA) and two Carotid Wallstents (8 × 29 mm and 8 × 21 mm, Boston Scientific, Marlborough, MA, USA). The procedure successfully restored patency of the right ICA (Figure 3D).

**Figure 4:**
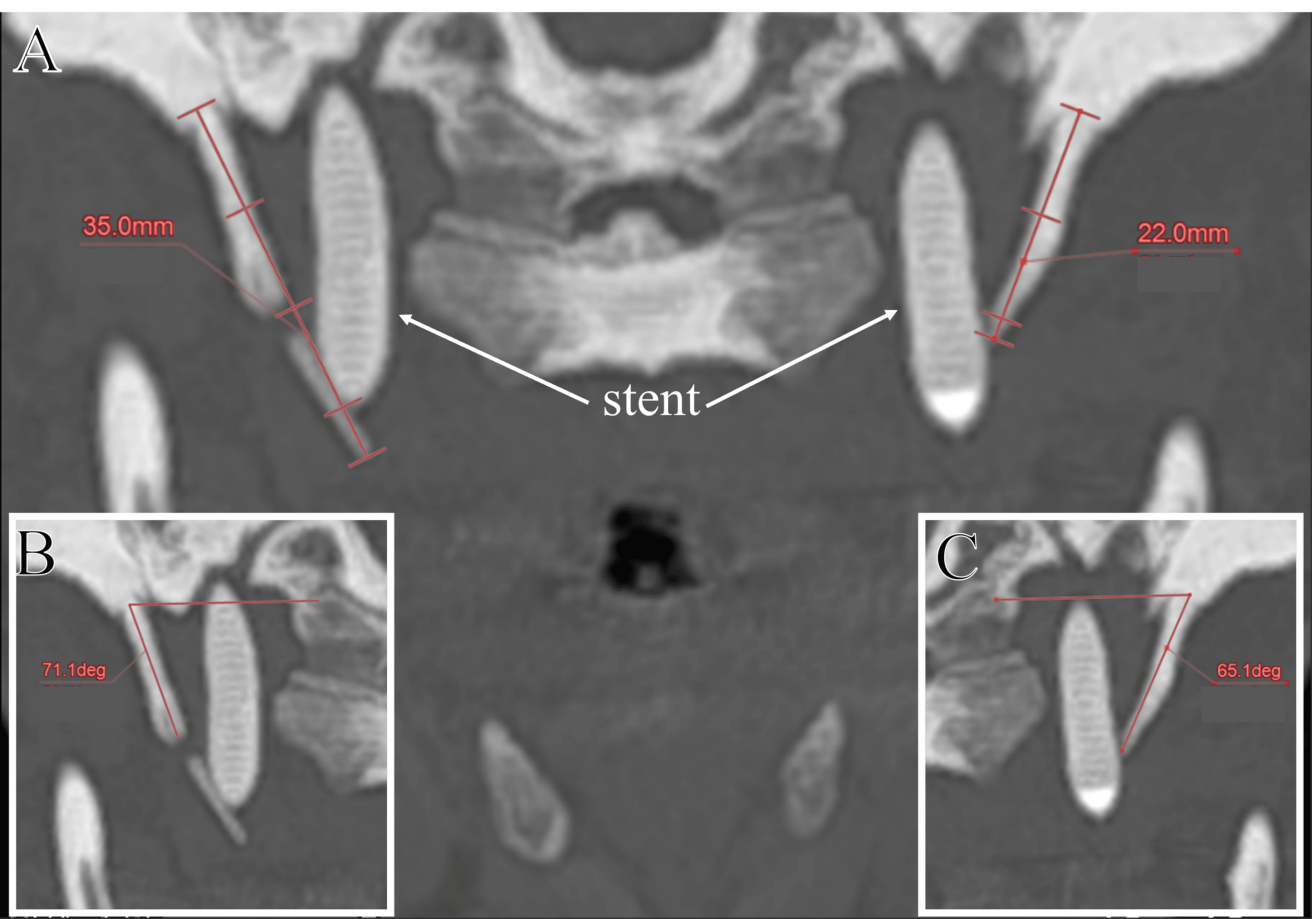
CT scans with maximum intensity projection (MIP) reconstruction before styloidectomies. Coronal plane image showing the SP lengths of 35 mm on the right and 22 mm on the left (A). The medial SP angulation measuring 71.1° on the right and 65.1° on the left, respectively (B-C). The right SP exhibits the morphological characteristics consistent with “calcification of the stylohyoid chain with segmentation”. SP: styloid process

**Figure 5:**
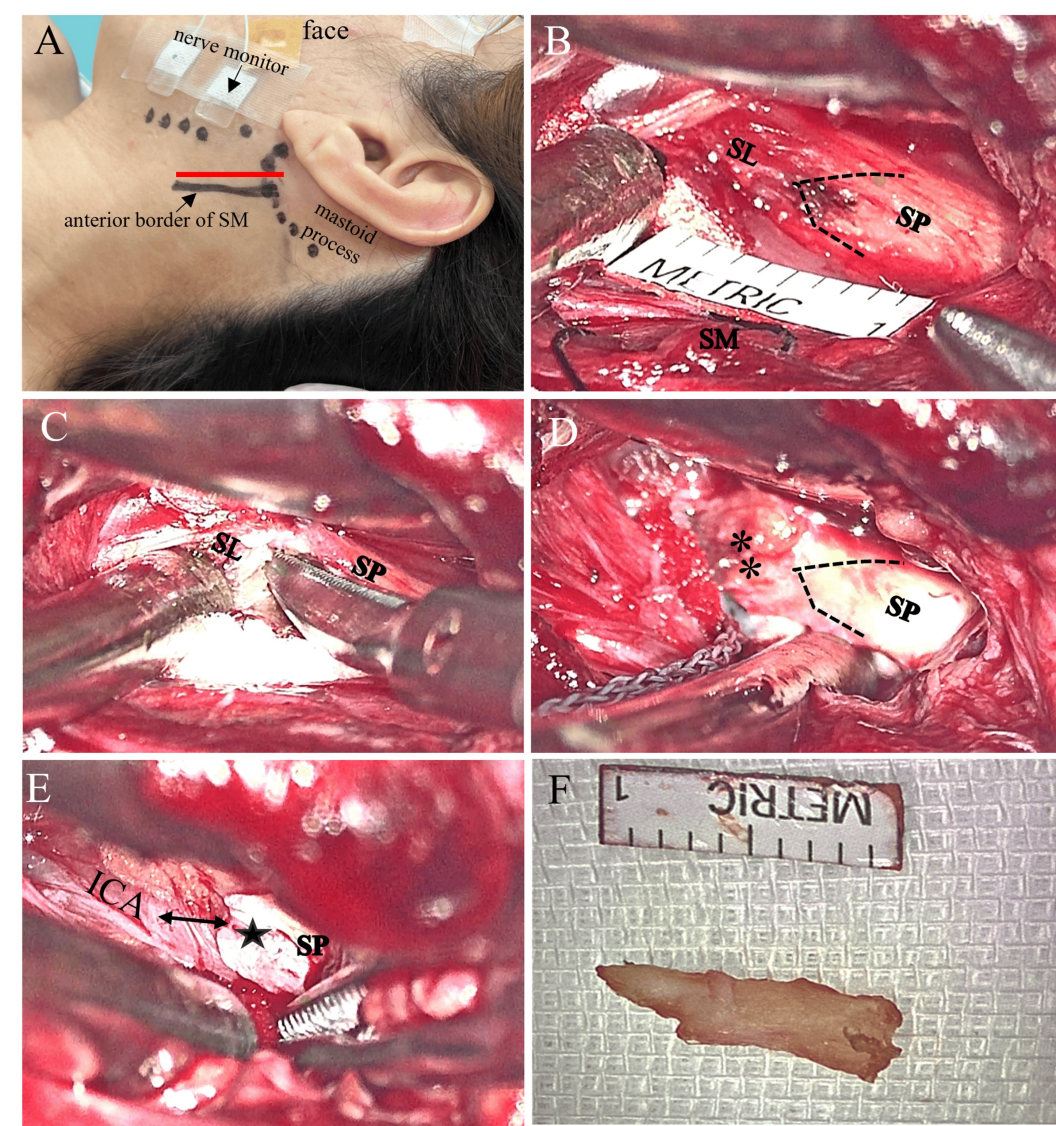
Intraoperative microscopic photographs showing the key procedural steps of the left styloidectomy. The patient was placed in the supine position with the neck slightly rotated to the right. Facial nerve monitoring was employed to minimize the risk of nerve injury. A skin incision approximately 4 cm in length (red line) was made posterior to the mandibular angle, extending downward along the anterior border of the sternocleidomastoid muscle (SM) (A). Upon deeper dissection, the SP, attached to the stylohyoid ligament (SL), was identified. Using a Doppler probe, the internal carotid artery (ICA) was confirmed to lie just beneath the SP. The dotted line indicates the outline of the SP tip (B). The connection between the SP and the SL was completely severed. Double asterisks indicate the resected edge of the SL (C). The body of the SP was fully exposed, and approximately 1 cm of the SP was removed using a rongeur (D). The stamp of the residual SP (star) was confirmed to be well separated (double-sided arrow) from the carotid sheath (ICA) (E). The excised SP tips measure approximately 1 cm in length (F). ICA: internal carotid artery; SL: stylohyoid ligament; SM: sternocleidomastoid muscle; SP: styloid process

**Figure 6:**
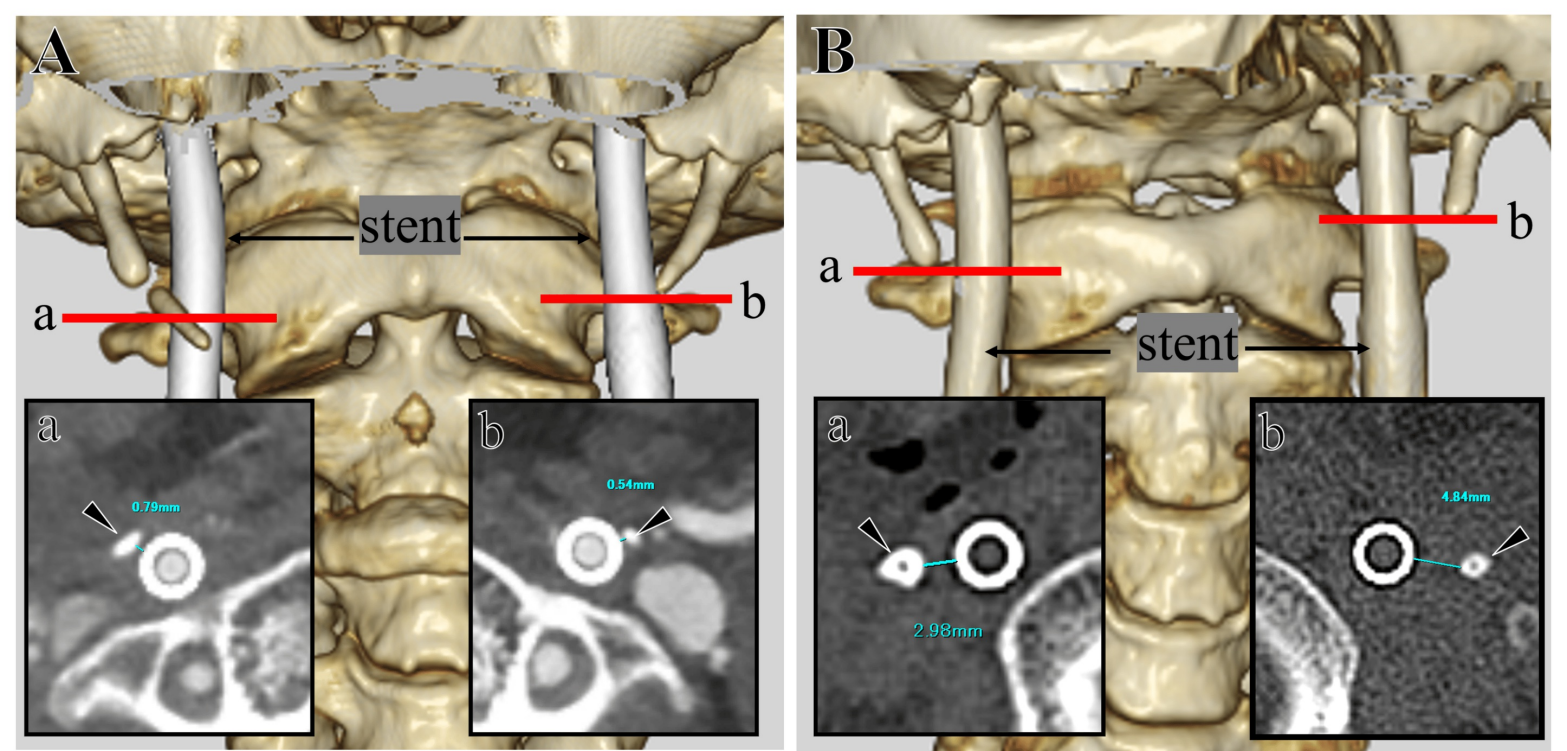
Three-dimensional (3D) reconstructed and axial CT imaging before and after bilateral styloidectomies. Preoperative 3D image showing the close proximity of the bilateral SPs to their respective ICAs (A). Preoperative axial images at the levels indicated by red lines demonstrate a close relation between the SPs (arrowhead) and stent-loaded ICAs, measuring 0.8 mm on the right (A-a) and 0.5 mm on the left (A-b). Postoperative 3D image after partial resection of both SPs (B). Postoperative axial images at the levels indicated by red lines demonstrating increased separation between the SPs (arrowhead) and stent-loaded ICAs, measuring 3.0 mm on the right (B-a) and 4.8 mm on the left (B-b). ICA: internal carotid artery; SP: styloid process

Postoperative CT imaging showed that the right SP measured 35 mm in length with a medial angulation of 71.1°, while the left SP measured 22 mm with a medial angulation of 65.1°.On both sides, the SP tips were in extremely close proximity to the ICA walls, with distances of less than 1 mm (Figure 4 and Figure 6A). Medial angulation was determined as the angle between the long axis of the SP and a line connecting the SP bases using maximum intensity projection (MIP) reconstruction images. The minimum distances between the SP tips and their respective ICAs were measured on axial bone window CT scans. All measurements were performed according to the method described by Tardivo et al. [7]. Three and six months after the second intervention, the patient underwent left and right transcervical styloidectomies, respectively, to eliminate the suspected mechanical compression on the bilateral ICAs (Figure 5).

**Figure 7:**
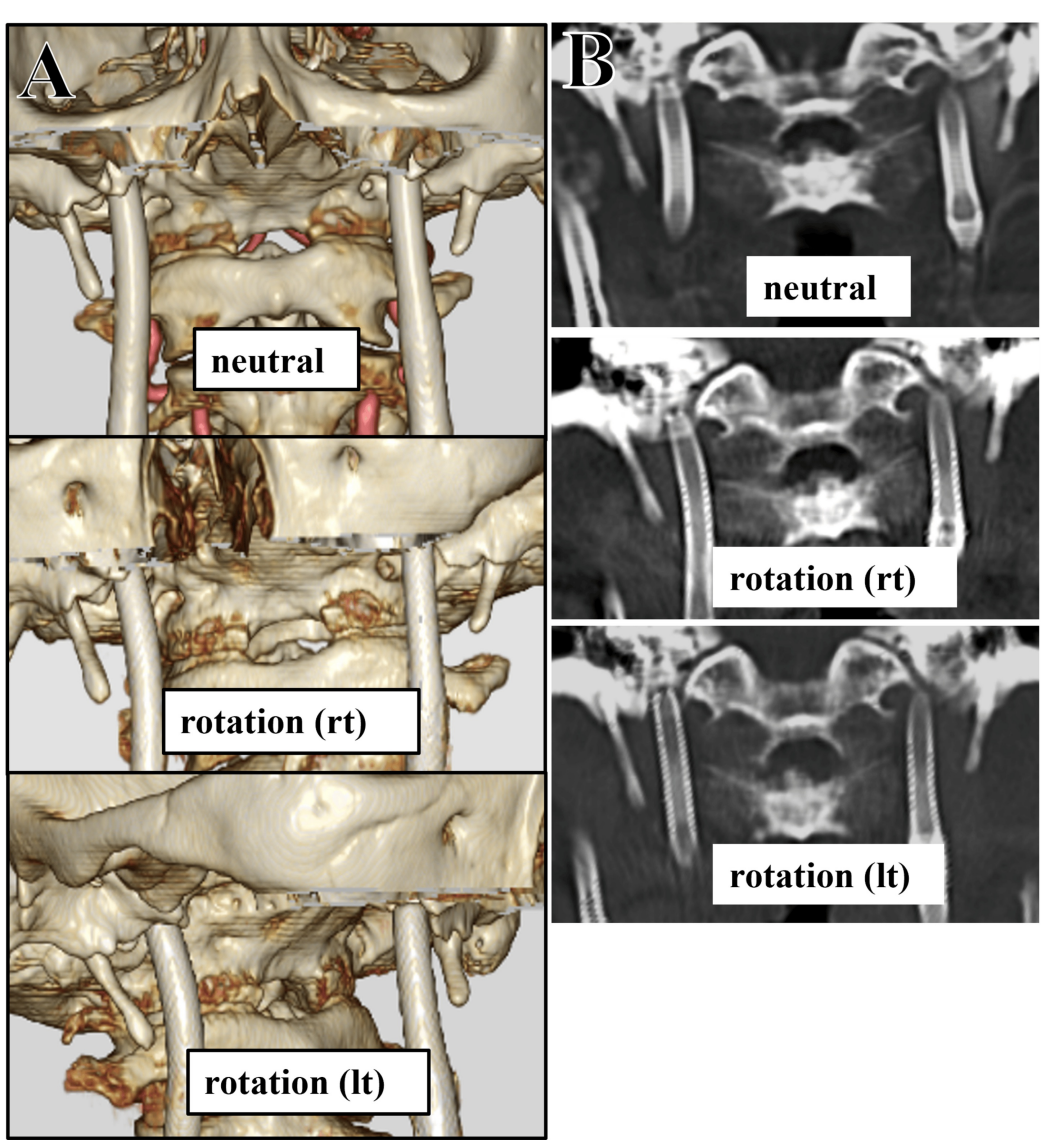
Three-dimensional reconstructed CT angiography (3D-CTA) and CT scans with maximum intensity projection (MIP) reconstruction with dynamic head rotation following bilateral styloidectomies. 3D-CTA images showing the spatial relationship between the SPs and ICAs in different head positions (upper: neutral, center: rightward rotation, lower: leftward rotation). The anatomical gap between each SP and its respective ICA is preserved in all positions (A). MIP CT reconstructions in dynamic head positions (upper: neutral, center: rightward rotation, lower: leftward rotation), again confirming that the distances between the bilateral SPs and their respective ICAs remain preserved in all positions (B). ICA: internal carotid artery; SP: styloid processes; rt: right; lt: left

Postoperative imaging confirmed that the bilateral SP tips were well separated from the ICAs, effectively resolving the compressive condition even during neck movement (Figure 6B and Figure 7). The patient’s postoperative course following bilateral styloidectomies was uneventful. She remained neurologically intact throughout her hospital stay and was discharged home with an NIHSS score of 0.

## Discussion

Anatomically, the SP is a bony prominence that extends downward from the temporal bone and is connected to the stylohyoid ligament [1]. In the general population, the SP measures between 20 mm and 30 mm in length [2, 11]. However, it can be abnormally long due to factors such as congenital variations, trauma, or inflammatory reactions [2, 3]. When the SP length exceeds 30 mm, it is traditionally classified as “elongated” [2, 6, 9, 15]. Additionally, the SL can undergo calcification, forming a stylohyoid chain that connects to the SP, potentially further increasing its length [3, 15]. Anatomical variations like elongated SPs and calcified stylohyoid ligaments are found in approximately 4-10% of the population [1-3]. However, those with anatomical variations do not always lead to clinical symptoms, with only 4% of them being symptomatic [1-3]. The SP and its associated ligaments are situated near neurovascular structures, including the lower cranial nerves, internal and external carotid arteries, and veins [1-3]. Although the normal SP does not directly compress these neurovascular structures, anatomical variations such as elongation, calcification of the associated ligament, and abnormal relationships to surrounding structures can lead to SP-related compression, resulting in clinical symptoms [5-9, 15].

Eagle syndrome and SCAS are clinically overlapping yet pathophysiologically distinct entities. Eagle syndrome typically occurs when an elongated SP impinges on nearby neurovascular or soft tissue structures, resulting in foreign-body sensation, craniofacial pain, and neuropathic or vascular symptoms [1-3]. In contrast, SCAS arises when a SP compresses the external or ICA, resulting exclusively in vascular manifestations, such as stroke or transient ischemic attack [5, 6]. While Eagle syndrome is traditionally defined by a SP length of 30mm or greater [2, 3], SCAS does not necessarily require a specific SP length for diagnosis [5, 6]. Based on this definition, the present case could be diagnosed as bilateral SCAS or as a combination of vascular Eagle syndrome and SCAS. Although both conditions are commonly associated with elongated SPs, SP length does not always correlate with clinical manifestations. Symptoms related to these conditions generally occur unilaterally, with bilateral presentations being exceedingly rare [10-14]. A unique feature of this case is the bilateral occurrence with asymmetric SP morphology, where one side was abnormally elongated while the other exhibited normal anatomical length.

The risk of vascular symptoms associated with the SP is closely correlated with its length, as an elongated SP may exert greater mechanical force on adjacent structures [2]. Consequently, CAD caused by a normal-length SP is rare; to date, only a few such cases, including the present one, have been reported in the literature [7-9]. Tardivo et al. proposed that factors other than SP length may also contribute to the pathogenesis of SP-related CAD [7]. These factors include steep medial angulation of the SP and reduced SP-ICA distance (Table 1) [7]. In the present case, the right SP measured 35 mm (elongated), while the left SP was normal in length at 22 mm. Despite the discrepancy in length, both SPs were in close proximity to their respective ICAs, with a distance of less than 1mm on each side. According to Tardivo et al., the average medial angulation of the SP in the normal population is 71.92°, and the typical SP-ICA distance is 6.08 mm (Table 1) [7]. Notably, the left SP in the present case, though of normal length, exhibited a steep medial angulation of 65.1°, possibly contributing to its close proximity to the ICA and the subsequent development of CAD. Recently, Osuki et al. reported a CAD case with remarkably similar morphological and clinical features [8]. Their patient also had a normally sized SP (21.9 mm) and underwent CAS followed by styloidectomy, as in our case. Although SP angulation was not measured in their report, they emphasized the pathophysiological significance of the SP’s close proximity to the ICA.

**Table 1:** Morphological parameters of styloid processes implicated in carotid artery dissection. CAD: carotid artery dissection; ICA: internal carotid artery; SP: styloid process

	Tardivo et al.[7]		Present case	
	CAD (n=10)	Normal control (n=31)	Right (elongated)	Left (non-elongated)
SP length (mm)	40.58	28.74	35	22
SP angle (°)	67.67	71.92	71.1	65.1
SP-ICA distance (mm)	1.67	6.08	< 1	< 1

In the present case, calcification of the SL likely contributed to elongation or chain formation on the right side, while the left side retained a congenital morphology. Thus, anatomical variations in SP morphology, including length, angulation, proximity to the ICA, and ligamentous calcification, may all play significant roles in the pathogenesis of CAD [7, 15]. This case highlights the importance of evaluating the overall anatomical configuration of the SPs, rather than relying solely on length, when assessing the risk of vascular complications such as CAD.

There is currently no consensus on the optimal treatment for SP-related CAD [8-14]. When CAD poses a risk of significant luminal stenosis or occlusion of ICA, interventional management such as CAS may be considered in addition to conservative antiplatelet or anticoagulant therapy [8, 9, 10-14]. However, due to the close anatomical proximity of the unresected SP to the ICA, persistent mechanical stress may cause recurrent ICA injury [6, 8, 9, 16, 17]. Among these ischemic complications, mechanical stent fracture is a serious post-procedural complication even after successful interventions, as it may result in vessel occlusion and severe ischemic events [6, 16, 17]. In light of this risk, stenting alone may be insufficient to prevent ischemic recurrence. Therefore, prophylactic SP resection may offer a more definitive treatment to prevent further ischemic events [8 -14, 16, 17]. Two common surgical routes exist for SP resection: intraoral and transcervical [1, 3, 9]. While the intraoral approach has the advantages of a shorter operative time and no external scarring, it offers limited surgical exposure and carries risks of major vessel injury and retropharyngeal infection. In contrast, the transcervical approach allows a better view and a safer access to the SP, with a lower risk of vascular complications [3, 9]. Nevertheless, it is associated with potential complications, including cranial nerve injury involving the glossopharyngeal, hypoglossal, or facial nerves, although the precise incidence has not been well documented [3, 9]. Long-term outcomes after styloidectomy appear favorable. While the recurrence rate after conservative antiplatelet or anticoagulant therapy - with or without CAS - is reported to exceed 20%, the recurrence rate following styloidectomy is approximately 1% [9]. In this case, the patient had a SP of normal length (22 mm) on one side and a slightly elongated one (35 mm) on the other, raising concerns about incomplete resection via the intraoral route. Considering the anatomical characteristics and the surgeon’s experience, the transcervical approach was performed to ensure an adequate resection of the SPs on both sides in this patient.

Nonetheless, further studies are needed to more comprehensively assess the safety profile and long-term efficacy of styloidectomy for patients with SP-related CAD.

## Conclusions

This case highlights the diagnostic and therapeutic challenges posed by vascular conditions associated with anatomical variations of the SP, such as Eagle syndrome or SCAS. While SP length has traditionally been emphasized in diagnosis, our findings suggest the importance of additional clinical and morphological features in identifying SP-related CAD. Key indicators include: (1) recurrent CAD in young individuals without conventional cardiovascular risk factors; (2) radiological evidence of close anatomical proximity between the SP and the ICA; and (3) steep medial angulation of the SP. These factors can support diagnosis even when the SP is of normal length.

Although treatment strategies for SP-related CAD remain controversial, this case demonstrates the potential efficacy of CAS. Additionally, prophylactic SP resection should be considered for definitive prevention of recurrence when CAS alone proves insufficient. Given that CAD involving a SP of normal length may be underrecognized, increased awareness of SP anatomical variations beyond length is crucial. We emphasize the importance of recognizing SP anatomical variations beyond length and tailored therapeutic approaches in managing this rare but clinically significant vascular disorder.
